# Mobile Phone and Web-based Cognitive Behavior Therapy for Depressive Symptoms and Mental Health Comorbidities in People Living With Diabetes: Results of a Feasibility Study

**DOI:** 10.2196/mental.5131

**Published:** 2016-05-31

**Authors:** Janine Clarke, Judith Proudfoot, Howard Ma

**Affiliations:** ^1^ Black Dog Institute Randwick Australia; ^2^ School of Psychiatry UNSW Australia Sydney Australia

**Keywords:** diabetes, depression, Internet interventions, eHealth, CBT

## Abstract

**Background:**

Depression is often comorbid with diabetes; however, undertreatment of depressive symptoms in people affected is common.

**Objective:**

We studied preliminary acceptability and effectiveness of a fully automated, mobile phone, and web-based public health intervention, myCompass, for reducing depressive symptoms and improving mental health comorbidities in people with diabetes.

**Methods:**

In this single-group feasibility study, 89 volunteers with type 1 (n=34) or type 2 (n=55) diabetes and at least mild depressive symptoms used myCompass for 7 weeks. Web-based measures of depressive and anxious symptoms, functional impairment, diabetes-specific variables, and user satisfaction were completed at baseline, postintervention, and 3-month follow-up.

**Results:**

Retention rates were 54% (n=48) at postintervention and 36% (n=32) at follow-up. Depressive symptoms were significantly improved at postintervention (*P*<.001; within-group effect size d=1.05), with gains persisting at follow-up. Mental health comorbidities, including anxiety (*P*<.001), functioning (*P*<.001), and diabetes-specific distress (*P*<.001), also showed significant and sustained improvement. Satisfaction with myCompass was high, with convenience and ease of program use, and relevance of program content rated positively by participants.

**Conclusions:**

The myCompass program shows promise as an acceptable and effective treatment for depression and comorbid mental health problems in people with diabetes. The program is broadly available, free to use, and may benefit patients with diabetes who do not access services and/or wish to manage their mental health themselves. Replication of these findings in a controlled study is warranted.

## Introduction

Depression is frequently comorbid with diabetes, affecting 10% to 30% of the estimated 415 million people with the disease [1], and contributes independently to poorer daily management of diabetes regimen tasks or “self-care,” higher rates of microvascular and macrovascular complications, elevated health service costs, and increased mortality [2]. Evidence-based treatments for comorbid depression are available, including face-to-face cognitive behavior therapy (CBT) and antidepressant medication, and evidence supports the utility of these approaches for improving self-management of diabetes and glycemic control [3,4]. Psychotherapeutic approaches are particularly beneficial as they lack the side effects of medication [5], and there is evidence that treatment gains in CBT are maintained for up to 1 year past cessation of treatment [6].

The existence of effective treatments means that the adverse consequences of depression for people with diabetes are not inevitable [3,7]. Nevertheless, it is estimated that only one-third of people with diabetes and depression receive appropriate treatment for both disorders [8]. In the primary care setting, where medical support for most patients with diabetes is provided, only a minority of patients who screen positive accept a referral for face-to-face support [9]. At the same time, barriers to help seeking, including lack of psychological services (especially in rural and remote areas), financial cost, concerns about confidentiality and stigma, and time and lifestyle constraints [10], compromise access to satisfactory care for many patients. There is, therefore, considerable opportunity to improve diabetes management and to intervene with disease progression by increasing patient access to effective depression treatments that reduce structural and patient barriers to care and offer the advantages of user privacy and 24-hour availability.

The Internet is a popular, clinically effective and cost-efficient means of increasing access to empirically supported psychological treatments, and diabetes-specific Internet-delivered programs targeting depressive symptoms are available [11-13]. Nevertheless, a public health–focused intervention that is delivered through the Internet and generic in its therapeutic content (ie, capable of targeting depressive symptoms across a range of physical health conditions) may have a number of benefits over disease-specific approaches. In addition to facilitating broader treatment reach, a generic treatment program delivered through the Internet would assist the increasing number of individuals experiencing multimorbidity, for whom depression co-occurs with somatic symptoms of multiple illnesses (eg, diabetes, heart disease, hypertension, and kidney disease [14,15]). Related to this, a generic intervention is likely to be more efficiently and easily disseminated in the primary care setting, where treatment of multimorbidity and undifferentiated physical and mental health symptoms are particularly relevant.

We have previously demonstrated the effectiveness of a fully automated public health intervention, myCompass, for improving symptom and functional outcomes for people with mild-to-moderate depression, anxiety, and stress [16,17]. Grounded in CBT, myCompass provides 24/7 access to psychotherapeutic support and real-time monitoring of thoughts, feelings, and behaviors using mobile phone and Web technology. Compared with active control and waitlist conditions, use of myCompass for 7 weeks reduced symptoms to within the near-normal range, with benefits persisting for 3 months [16].

This study was largely exploratory and aimed to assess initial acceptability and effectiveness for a larger randomized controlled trial of myCompass as an intervention for depression in diabetes. Depression and anxiety often co-occur [18,19], and as both increase the functional burden of chronic disease (eg, more days of missed work; [20]), we expected that myCompass would have important collateral advantages for patients with diabetes over and above the amelioration of depressive symptoms. Specifically, we hypothesized that people with diabetes and at least mild depression would show a pattern of symptom and functional gains consistent with the improvements observed for participants in our earlier trial.

Our secondary aim was to examine whether the benefits of myCompass extended beyond affective symptoms and general functional impairment to include disease-specific cognitions that correlate with depression and mediate diabetes outcomes. In particular, we were interested in whether the intervention would increase diabetes self-efficacy, that is, people’s confidence in their ability to perform diabetes self-care tasks [21] and reduce diabetes-related distress, that is, a person’s emotional adjustment to the various chronic stressors of diabetes (eg, fear of complications, feelings of isolation, distress associated with insulin, and frustration with daily self-care; [22]). Both diabetes self-efficacy and diabetes-related distress are clinically relevant variables with direct links to performance of diabetes regimen tasks (including blood glucose monitoring, healthy eating, exercise, taking medication, and foot care) and glycemic control [21-23]. Whereas previous studies have documented the benefits for these variables of disease-specific interventions (eg, [11,24]), we wanted to learn whether improvement in diabetes-specific cognitions might be possible using a CBT-based public health intervention with no disease-relevant content.

## Methods

### Recruitment

Participants were recruited nationally between March and November 2013 via advertisements placed on social media (Facebook and Twitter); Websites of the Black Dog Institute and diabetes associations in Victoria, New South Wales, and Queensland and in radio and print media. Those interested were invited to visit a study-specific Website to access information about the study, a Web-based consent form, and a screening survey.

Eligibility criteria included: diagnosed with type 1 (T1D) or type 2 (T2D) diabetes by a general practitioner (GP) or endocrinologist; Australian resident aged 18 to 75 years; has access to the Internet via mobile phone and computer; has a valid email address; reports symptoms of at least mild depression (score > 4 on the Patient Health Questionnaire (PHQ-9; [25]); and has no previous experience with myCompass. Individuals who endorsed psychotic symptoms on the Psychosis Screening Questionnaire (PSQ; [26]) were excluded from the study.

### Procedure and Design

A within-subject, prepost design was used for this study that was conducted entirely online. Eligible participants completed a baseline questionnaire and were automatically registered with the myCompass program. Because existing efficacy data for myCompass are based on a 7-week intervention period [16], study participants were provided access to the full program for 7 weeks and encouraged to use it *ad libitum* during this time. At the end of 7 weeks and again at week 20, participants completed the Web-based postintervention and follow-up questionnaires, respectively.

The study was approved by the University of New South Wales’ Human Research Ethics Committee (HREC12616) and registered with the Australian and New Zealand Clinical Trials Registry (ACTRN12613000172707).

### Intervention

**Figure 1:**
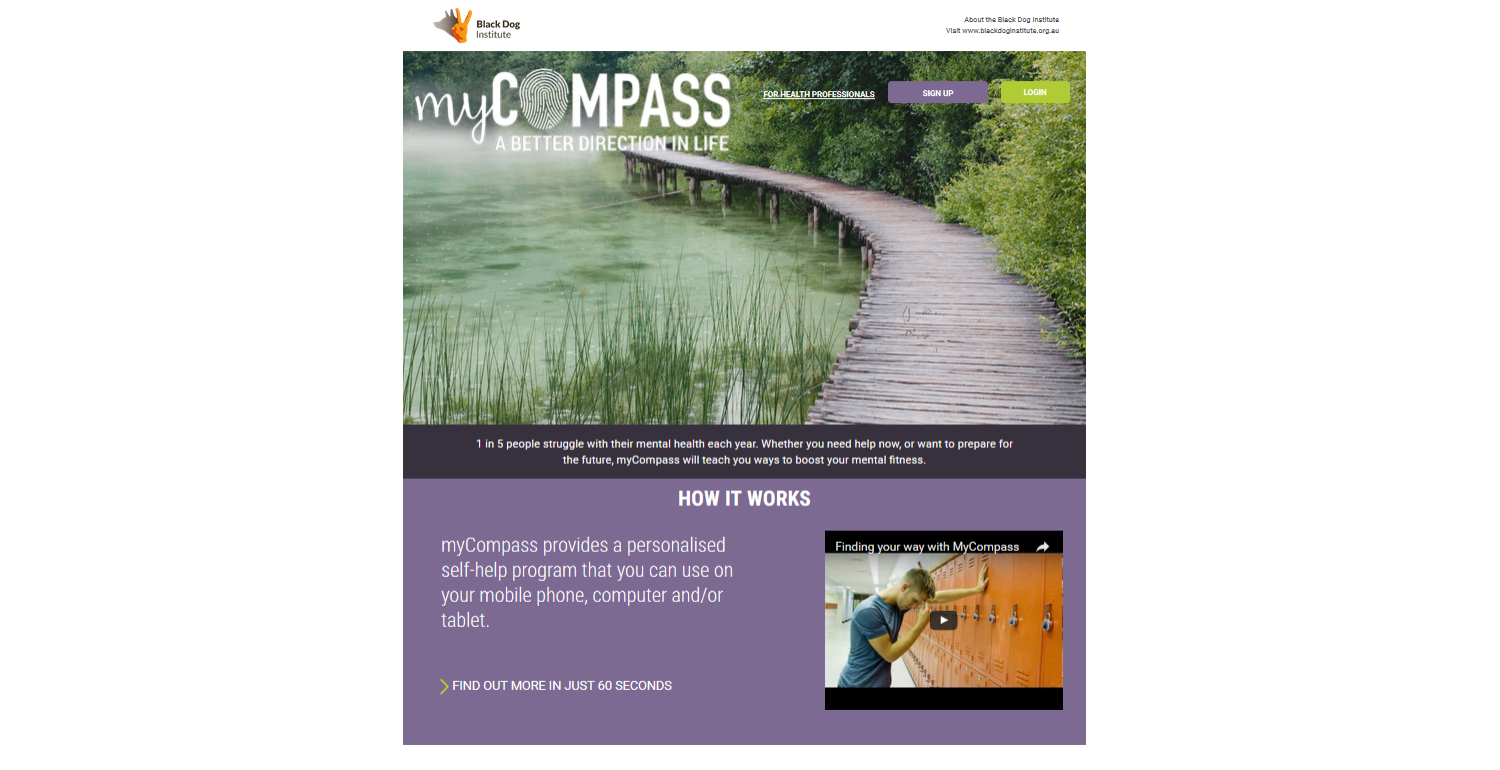
Screenshot of myCompass landing page (1).

**Figure 2:**
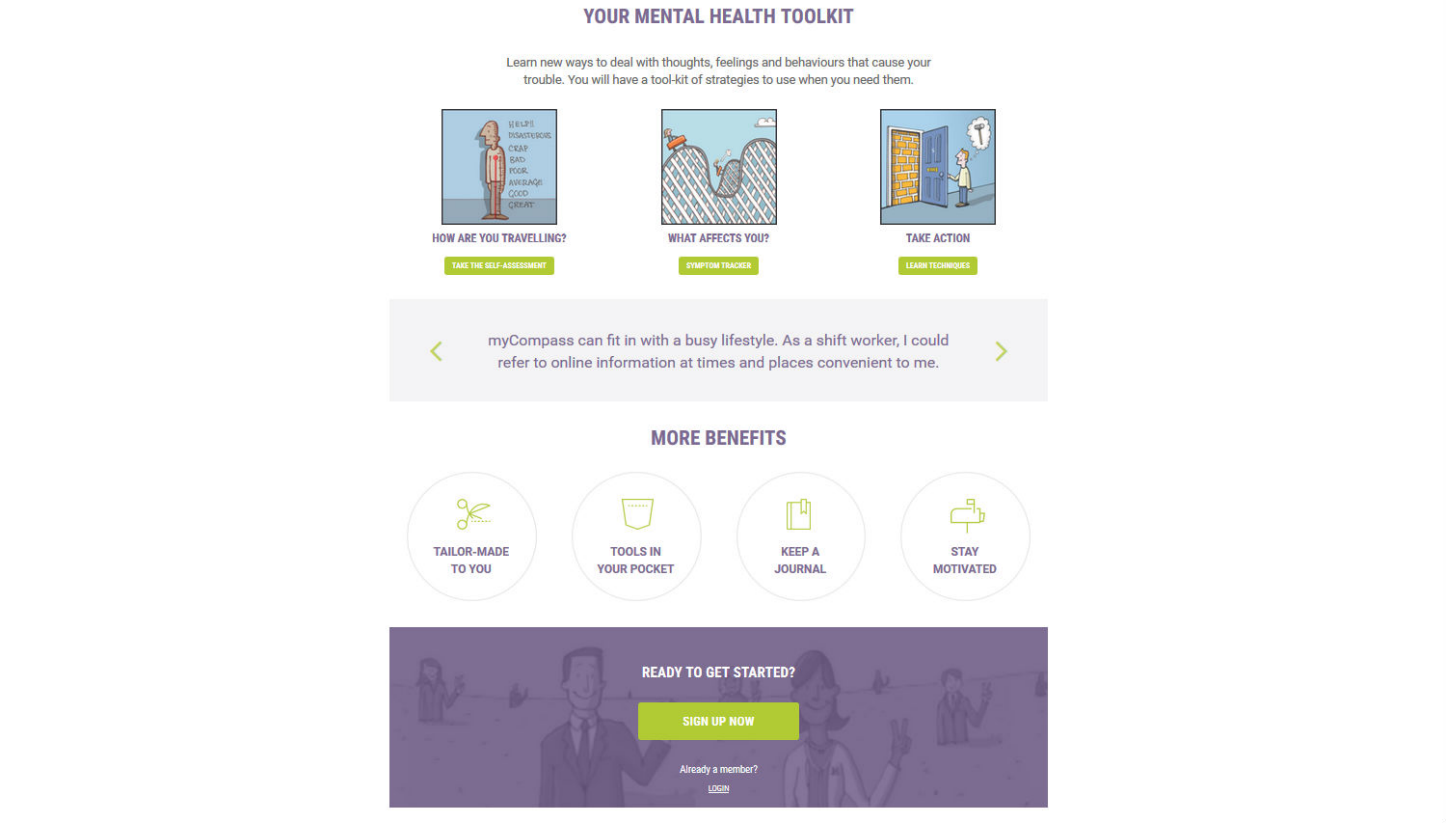
Screenshot of myCompass landing page (2).

**Figure 3:**
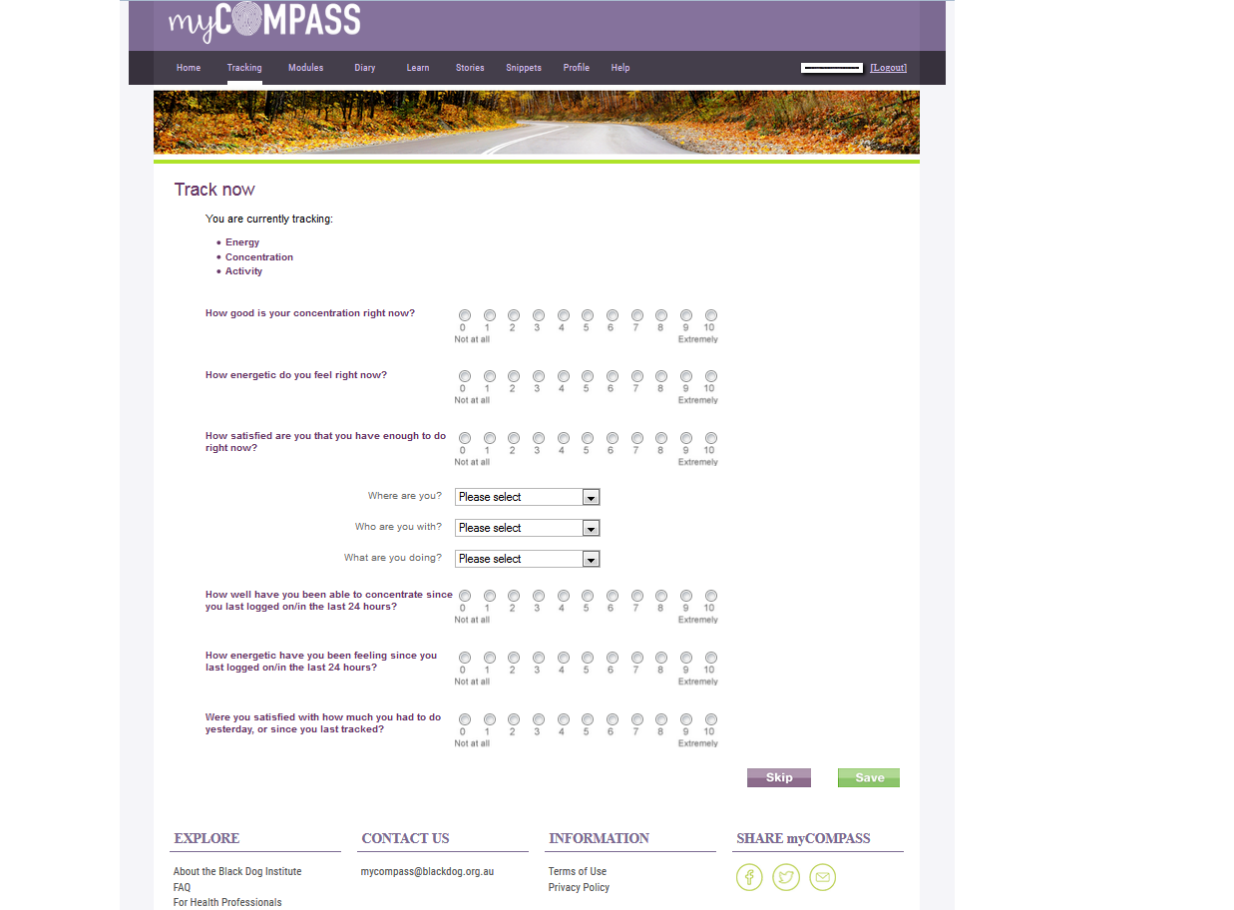
Screenshot of myCompass self-monitoring.

**Figure 4:**
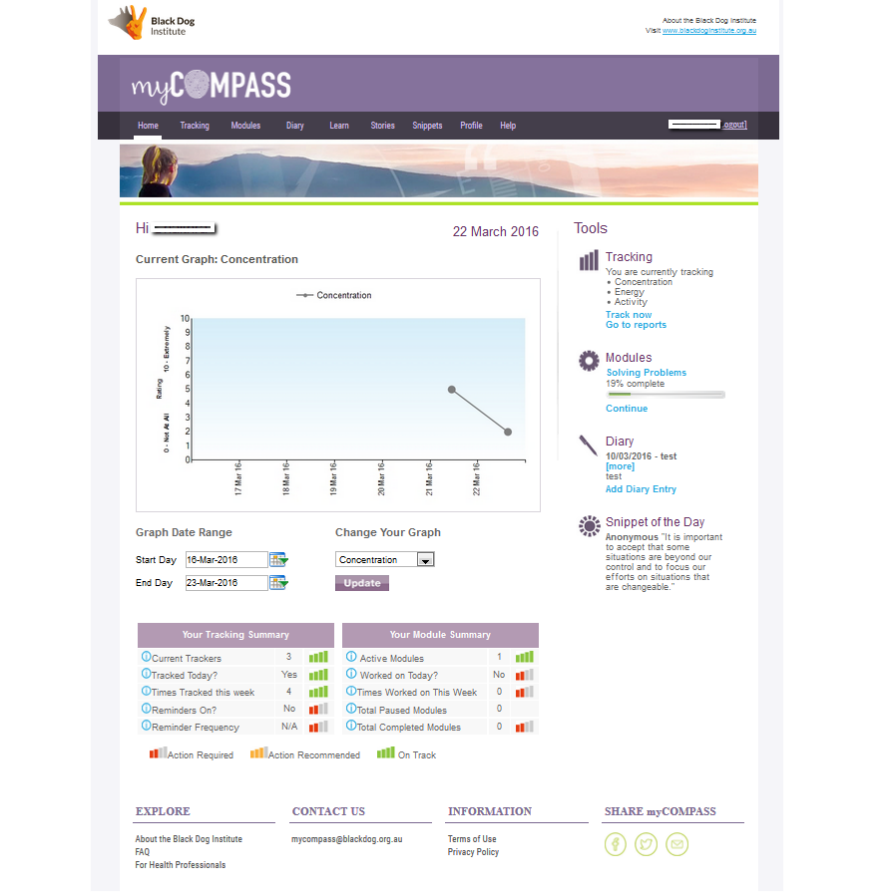
Screenshot of myCompass user home page.

**Figure 5:**
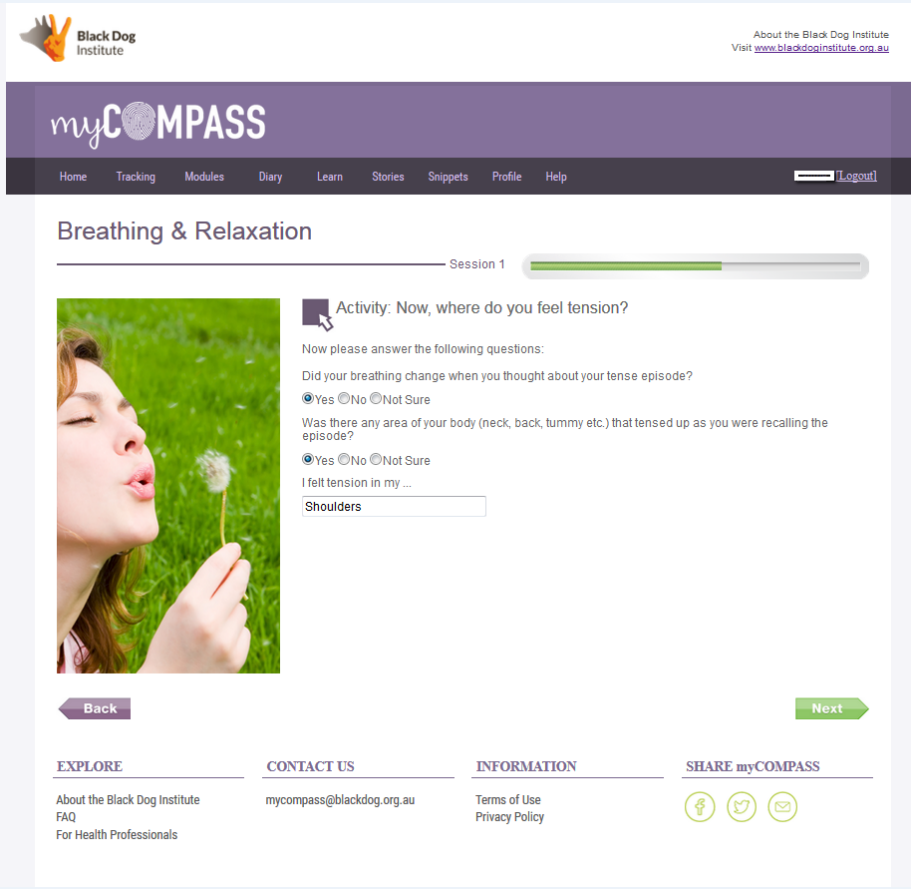
Screen from myCompass Breathing and Relaxation module.

The myCompass program [27] is a fully automated public health intervention (no therapist input) that is accessible from any Internet-enabled mobile phone, tablet, or computer (see Figures 1-5). The program assesses user symptoms and provides a personalized intervention that facilitates round-the-clock self-monitoring of moods and behaviors (via mobile phone, tablet, or computer) and provides interactive evidence-based learning modules (via tablet and computer). Each module contains 3, 5- to 10-minute sessions, each with an assigned homework task. Users are encouraged to complete 1 module session per week, with the aim of completing 2 full modules during the intervention period.

In addition, users can schedule text messaging (short message service, SMS) or email reminders to facilitate self-monitoring; receive and print graphical feedback about their self-monitoring (including contextual information) on their mobile phone or computer (to monitor change and assist identification of triggers); and elect to receive helpful facts, mental health care tips or motivational statements by SMS text messaging or email. Registering to use the program is free, and users are not billed for the SMSs they receive. A detailed description of the myCompass intervention is provided in the study by Proudfoot et al [16].

Providing feedback to program users improves adherence with Web-based interventions [28], and so we enhanced the functionality of myCompass in this study to send automated and personalized email messages to participants (at 4 weeks) about their use of the program’s self-monitoring and module functions. Messages were designed to be motivating: infrequent users were reminded of the benefits of regular program use; and frequent users were encouraged to continue self-monitoring and explore additional program modules.

### Measures

Participants provided sociodemographic (age, gender, highest educational qualification, and employment status) and disease-related information (age at diagnosis, treatment modality, and diabetes complications status) at baseline. At each assessment point, participants completed the following standardized and psychometrically sound measures.

#### Primary Outcomes

Depression and anxiety were measured by the PHQ-9 [25] and the Generalized Anxiety Disorder 7-item scale (GAD-7; [29]), respectively. The scales are well validated [30], used widely as screening tools in primary care settings, and frequently included as outcome measures in studies of Web-based interventions [31,32]. Scores of 5, 10, and 15 are used as cutoff points for mild, moderate, and severe symptoms, respectively, on both the scales.

Mental health self-efficacy was assessed using the Mental Health Self-Efficacy Scale (MHSES; [17]), which measures people’s confidence in managing mental health issues using six 10-point Likert scale items. Scores are summed to obtain an overall measure, with higher scores indicating greater mental health self-efficacy.

The Work and Social Adjustment Scale (WSAS; [33]) measured the impact of mental health problems on daily functioning in 5 domains: work, social leisure activities, private leisure activities, home management, and personal relationships [33,34]. Scores range from 0 to 40, with higher scores indicating poorer adjustment.

#### Secondary Outcomes

Emotional adjustment to diabetes was measured using the 20-item Problem Areas in Diabetes scale (PAID; [35,36]) Items are rated on a 5-point Likert scale (0-4) and assess distress caused by treatment, food, diet, social support, and emotional issues. Total scores on the PAID are multiplied by 1.25 to yield a score within the range of 0 to 100, with scores greater than 40 indicating elevated diabetes-related distress [37].

Diabetes self-efficacy was measured using the 8-item Diabetes Self-efficacy Scale (DSES; [38]), which asks about people’s beliefs in their ability to perform a range of diabetes self-care activities. Scores on the DSES are the average response to scale items and range from 1 to 10, with higher scores indicating greater diabetes self-efficacy.

The Summary of Diabetes Self-Care Activities Scale (DSCAS; [39]) measures diabetes self-management across 4 regimen areas: diet, exercise, blood glucose testing, and foot care. Mean scores are calculated for each area and range between 0 and 7, with higher scores representing better self-care.

Glycemic control was measured indirectly by asking participants to report on recent symptoms of hypoglycemia (eg, headaches, light-headedness, weakness) and hyperglycemia (eg, increased thirst, dry mouth, decreased appetite) using the scales developed by Piette [40]. Scores for each symptom domain range from 0 to 7, with higher scores reflecting more symptoms.

A combined quantitative and qualitative method was used to investigate participant’s views of the program and its utility. The postintervention questionnaire included 8 items that asked users to rate (0-4) their satisfaction with the usability, content, flexibility, and functionality of myCompass [16]. Scores ranged between 0 and 32 with higher scores reflecting a more positive user experience. In addition, data indicating extent of user engagement with myCompass were extracted from the program, including frequency of logins, number of modules completed, and self-monitoring frequency.

Quantitative data were supplemented with information obtained from brief telephone interviews with a subset of participants about their experience with the myCompass program. A standard “sampling to saturation” recruitment method yielded a total of 18 interviews that were audiotaped and later transcribed to identify emergent themes.

### Statistical Analyses

Statistical analyses were completed using SPSS 21.0 software. Descriptive statistics were calculated for baseline data, and independent *t*-tests, chi-square tests, and bivariate correlations were used, as appropriate, to examine: (1) differences between people with T1D and T2D on the demographic and disease-related characteristics; (2) relations between demographic and disease-related variables and scores on the outcome measures (to identify potential covariates); and (3) possible biases in study attrition.

Postintervention and follow-up treatment effects were examined based on the intention-to-treat sample using linear mixed modeling (LMM; [41]). In LMM, incomplete cases are included in the analysis, and all available data are used to obtain parameter estimates. Restricted maximum likelihood was used to estimate model parameters, and Satterthwaite’s approximation was used to obtain degrees of freedom. Analyses assumed a compound symmetric structure [42] and included all identified covariates in addition to baseline scores on the outcome variable of interest. Significant effects were tested using separate contrasts to compare scores at baseline and postintervention, and postintervention and follow-up. All effects were tested at the *P*<.05 level, with adjustment for the number of contrasts.

Cohen’s *d* was calculated to obtain estimates of treatment effects using estimated marginal means for within-group changes on all outcome measures (based on the pooled standard deviation—SD).

## Results

### Participants

Of the 161 people who consented to screening, 91 were eligible to participate (Figure 2). Reasons for ineligibility included: screening questionnaire incomplete (*n=*26, 38%); no Internet-enabled mobile phone (*n=*20, 29%); symptoms in the normal range (*n=*7, 10%); psychotic symptoms (*n=*3, 4%), used myCompass previously (*n=*5, 7%), not an Australian resident (*n=*4, 6%), no diabetes (*n=*2, 3%), and no mobile phone (*n=*2, 3%). Two people subsequently withdrew from the study, and their data were excluded from the analyses.

Demographic and disease-related data for the study participants are summarized in Table 1. Most participants had T2D (*n*=55, 61.8%) were female (*n*=62, 69.7%), tertiary educated (*n*=41, 46.1%), married (*n*=46, 51.7%), employed at least part-time (*n*=57, 64%), nonsmoking (*n*=79, 88.8%), with a mean age of 48 years (*SD*=12 years). Mean age of diabetes onset differed significantly for people with T1D and T2D, being 18 years (*SD*=12 years) and 52 years (*SD*=10 years), respectively [*t* (87)=−5.45, *P*<.001]. Insulin therapy was more common in people with T1D [*X*^2^(1)=42.91, *P*<.001], and tablet therapy was more common in T2D [*X*^2^(1)=42.71, *P*<.001]. Half of the sample reported no diabetes-related complications (*n*=45, 50.6%), with diabetes-related eye, health, and sexual problems being the most frequently reported complications for both T1D and T2D participants.

**Table 1:** Demographic and disease-related characteristics.

Demographic characteristics		Type 1 diabetes (*n*=34)	Type 2 diabetes (*n*=55)	
*Age* (*mean, SD*^a^)		39.91 (11.72)		52.78 (10.21)
*Gender (n, %)*				
	Male	8 (24)		19 (35)
	Female	26 (76)		36 (65)
*Marital status (n, %)*				
	Single	9 (26)		9 (16)
	Married	16 (47)		30 (55)
	Other	9 (26)		16 (29)
*Educational level (n, %)*				
	Secondary school or lower	8 (24)		6 (11)
	Trade certificate or diploma	8 (24)		26 (47)
	University undergraduate degree or higher	18 (52)		23 (42)
*Employed (n, %)*		24 (71)		33 (60)
*Treatment type (n, %)* ^b^				
	Insulin^c^	34 (100)		17 (31)
	Tablets	3 (9)		44 (80)
	Diet or exercise	6 (18)		33 (60)
*Age of onset of diabetes* (*mean, SD*)^c^		18.4 (11.8)		52 (10.2)
*Diabetes complications (n, %)* ^b^				
	Eye problems	7 (21)		14 (26)
	Kidney problems	0 (0)		3 (6)
	Heart problems	4 (12)		11 (20)
	Foot problems	3 (9)		11 (20)
	Sexual problems	3 (9)		12 (22)
	None	21 (62)		24 (44)

^a^SD: standard deviation.

^b^Participants can select more than one option.

^c^Groups differ, *P*<.001.

Table 2 summarizes baseline scores on the outcome measures. When the recommended cutoffs were applied, depressive symptoms were in the moderate range (*M*=12.79, *SD*=4.7; [25]), anxiety symptoms were in the mild range (*M*=9.48, *SD*=4.04; [29]), and scores on the WSAS indicated significant functional impairment (*M*=17.44, *SD*=8.41; [33]). Scores on the diabetes (*M*=5.57, SD=2.1) and mental health (*M*=30.14, *SD*=11.11) self-efficacy measures indicated moderate levels of confidence in managing diabetes regimen demands and mental health issues, respectively, and diabetes-related distress was elevated (*M*=52.71, *SD*=18.28; [37]). Overall, symptoms of hypoglycemia (*M*=2.14, *SD*=1.37) and hyperglycemia (*M*=2.70, *SD*=1.47) were infrequent, and diabetes self-care was variable, with adherence greatest for blood glucose monitoring (*M*=4.32, *SD*=2.75) and poorest for foot care (*M*=1.90, *SD*=2.05).

**Table 2:** Observed scores on the outcome measures at baseline, postintervention, and follow-up.

			Baseline (*n*=89)		Postintervention (*n*=48)		Follow-up (*n*=32)	
		Mean	SD^a^	Mean	SD	Mean	SD
*Primary outcomes*								
	Depression (PHQ-9^a^)		12.79	4.70	7.94	4.20	9.03	4.51
	Anxiety (GAD-7^a^)		9.48	4.04	6.93	3.34	7.41	3.89
	Work and Social Functioning		17.44	8.41	14.63	8.22	13.06	7.53
	Mental Health Self-Efficacy		30.14	11.11	33.48	11.52	33.78	10.91
*Secondary outcomes*								
	Diabetes-distress (PAID^a^)		50.71	18.28	30.10	17.42	30.15	21.62
	Diabetes self-efficacy		5.57	2.08	6.12	2.10	6.23	1.99
	Diabetes self-care							
		Foot	1.90	2.05	2.33	2.34	2.45	2.48
		Diet	3.63	1.55	3.74	1.36	3.85	1.32
		Blood glucose monitoring	4.32	2.75	4.45	2.67	4.56	2.35
		Exercise	2.19	2.27	2.05	2.02	2.20	2.25
	Glycemic control							
		Hypoglycemia	2.14	1.37	2.42	1.32	2.31	1.44
		Hyperglycemia	2.70	1.47	1.92	1.33	1.97	1.59

^a^SD: standard deviation; GAD: generalized anxiety disorder; PHQ-9: Patient Health Questionnaire; PAID: Problem Areas in Diabetes scale.

### Identification of Covariates

Participants with T1D reported greater diabetes self-efficacy [*t* (86)=4.47, *P*<.001], more regular blood glucose monitoring [*t* (85)=4.51, *P*<.001], fewer hypoglycemic symptoms [*t* (86)=−2.39, *P*=.019], and reduced depressive symptoms [*t* (87)= −2.11, *P*=.037], compared with T2D participants. Length of diagnosis was positively correlated with diabetes self-efficacy [*r* (89)=0.36, *P*=.001], negatively correlated with depressive symptoms [*r* (89)=−0.22, *P*=.01], and scores were higher for women than men on the hyperglycemia scale [*t* (86)=−2.77, *P*=.007]. The influence of identified covariates on study outcomes was controlled in subsequent tests of treatment effects.

### Study Attrition

The attrition rate for the total sample at postintervention was 46% (*n*=41) and 64% (n=57) at follow-up (Figure 1). Participants who did and did not complete the postintervention and follow-up assessments were indistinguishable at baseline on the basis of their demographic and disease-related characteristics and scores on the outcome measures.

### Postintervention Outcomes

Observed mean scores on the study outcomes at postintervention and follow-up are summarized in Table 2. Results of the LMM analyses for the primary study outcomes are summarized in Table 3, which show a consistent pattern of sustained improvement for depressive and anxious symptoms, mental health self-efficacy, and work and social functioning. Improvement was also observed for some secondary outcomes including diabetes-related cognitions, diabetes foot care, and hyperglycemic symptoms (see Table 4).

**Table 3:** Results of LMM^a^analyses for primary outcomes: test of repeat effect (time).

Effect			Contrast estimate	*df*	F, *t* statistic	*P*	95% CI
*Depression*							
	Time			2, 132	38.14	< .001	
	*Contrasts*						
		Baseline vs postintervention	4.68	1, 127	8.30	< .001	3.31-6.04
		Postintervention vs follow-up	−1.28	1, 127	−1.76	.081	−3.0- 0.48
*Anxiety*							
	Time			2, 135	16.46	< .001	
	*Contrasts*						
		Baseline vs postintervention	2.51	1, 131	5.36	< .001	1.38-3.65
		Postintervention vs follow-up	−0.60	1, 129	−1.02	.311	−2.04- 0.83
*Work and Social Functioning*							
	Time			2, 132	12.96	< .001	
	*Contrasts*						
		Baseline vs postintervention	3.33	1, 129	3.87	< .001	1.25-5.40
		Postintervention vs follow-up	1.18	1, 125	1.07	.289	−1.50- 3.87
*Mental Health Self-Efficacy*							
	Time			2, 134	7.56	.001	
	*Contrasts*						
		Baseline vs postintervention	−4.52	1, 130	−3.39	.001	−7.75- −1.29
		Postintervention vs follow-up	0.10	1, 129	0.06	.955	−4.00- 4.19

^a^LMM: linear mixed modeling.

**Table 4:** Results of LMM analyses for secondary outcomes: test of repeat effect (time).

Effect				Contrast estimate	*df*	F, *t* statistic	*P*	CI
*Diabetes distress*								
	Time				2, 131	88.58	< .001	
	*Contrasts*							
		Baseline vs postintervention		20.51	1, 128	11.51	< .001	16.20-24.82
		Postintervention vs follow-up		0.15	1, 125	0.07	.947	−5.33- 5.64
*Diabetes self-efficacy*								
	Time				2, 128	5.96	.003	
	*Contrasts*							
		Baseline vs postintervention		−0.47	1, 125	−2.80	.006	−0.88- −0.06
		Postintervention vs follow-up		−0.09	1, 123	−0.40	.692	−0.62- 0.44
*Diabetes self-care*								
	*Foot*							
		Time			2, 128	4.09	.019	
		*Contrasts*						
			Baseline vs postintervention	−0.60	1, 126	−2.42	.017	−1.21- −0.00
			Postintervention vs follow-up	−0.03	1, 121	−0.08	.933	−0.78-0.73
	*Diet*							
		Time			2, 133	0.28	.760	
		*Contrasts*						
			Baseline vs postintervention	0.03	1, 131	0.20	.844	−0.33 -0.39
			Postintervention vs follow-up	−0.13	1, 128	−0.72	.476	−0.59 - 0.32
	*BGT* ^a^							
		Time			2, 126	0.79	.458	
		*Contrasts*						
			Baseline vs post intervention	−0.15	1, 125	−0.85	.399	−0.58 - 0.28
			Postintervention vs follow-up	−0.08	1, 119	−0.38	.708	−0.63 - 0.46
	*Exercise*							
		Time			2, 131	0.44	.647	
		*Contrasts*						
			Baseline vs postintervention	0.21	1, 129	0.89	.377	−0.36 - 0.79
			Postintervention vs follow-up	−0.21	1, 125	−0.70	.483	−0.93 - 0.51
*Physical symptoms*								
	*Hypoglycemia*							
		Time			2, 89	1.52	.224	
		*Contrasts*						
			Baseline vs postintervention	−0.32	1, 90	−1.59	.116	−1.00 - 0.36
			Postintervention vs follow-up	0.02	1, 82	0.10	.924	−0.85 - 0.90
	*Hyperglycemia*							
		Time			2, 130	12.99	< .001	
		*Contrasts*						
			Baseline vs postintervention	0.80	1, 128	4.79	< .001	0.40-1.21
			Post intervention vs follow-up	−0.18	1, 124	−0.86	.389	−0.70 -0.33

^a^BGT: blood glucose testing.

Within-group effect sizes at postintervention and follow-up ranged between moderate and large for the measures of depression and mental health comorbidities (Table 5). Effect sizes for the diabetes self-care and hypoglycemia and hyperglycemia scales were small, an exception being the moderate effect observed for diabetes foot care.

**Table 5:** Within-group effect sizes (Cohen’s *d*) at postintervention and follow-up.

Study outcome			Postintervention (*d*)	Follow-up (*d*)
*Primary outcomes*				
	Depression		1.05	0.74
	Anxiety		0.68	0.48
	Work and Social Functioning		0.40	0.57
	Mental Health Self-Efficacy		0.40	0.40
*Secondary outcomes*				
	Diabetes Distress (PAID^a^)		1.15	1.04
	Diabetes Self-Efficacy		0.22	0.27
	Diabetes Self Care			
		Foot	0.28	0.28
		Diet	0.02	0.07
		Blood glucose monitoring	0.06	0.09
		Exercise	−0.10	0.00
	Physical symptoms			
		Hypoglycemia	−0.24	−0.21
		Hyperglycemia	0.57	0.40

^a^PAID: Problem Areas in Diabetes scale.

### User Experience

Overall, mean ratings for the items assessing user experience were at or above the midpoint, suggesting that participants were largely satisfied with the program (Table 6). A total of 32 (67%) participants who returned postintervention questionnaires reported that they would recommend myCompass to other people with diabetes, and 28 (58%) indicated that they would happily use the program again.

**Table 6:** Descriptive statistics for items on the *myCompass* satisfaction survey (range 0-4).

No.	Question (*n*=48)	*Mean*	*SD* ^a^
1	myCompass was easy to use	2.93	1.10
2	myCompass was convenient to use	2.96	1.10
3	The information was easy to understand	2.91	1.30
4	The program kept my interest and attention	2.27	1.18
5	The program helped improve my stress, low mood, and/or anxiety	2.50	0.91
6	The program taught me skills that will help me handle future problems	2.42	1.14
7	The program has helped me to feel more in control of my stress, low mood, and/or anxiety	2.29	1.00
8	The program has helped me feel more in control of my diabetes	2.10	0.93

^a^SD: standard deviation.

On average, participants who returned postintervention questionnaires used the myCompass program 16 times (*SD*=20.47; range 0-84), self-monitored 26 times (*SD*=28.1; range 2-93), and completed almost 1 psychotherapeutic module (*M*=0.81, *SD*=1.05, range 0-4) during the 7-week intervention period.

Thematic analysis [43] was used to identify salient themes and ideas that emerged from interview responses regarding participant’s experience with myCompass. When asked what they liked about myCompass, interviewees generally agreed that the program was accessible (24/7) and convenient (mobile phone and computer) to use, with content that was engaging and useful for skill building and self-reflection.

"The fact that you could do it in your own time. You could go to it when you felt like it and when it was gonna [sic] do you the most good.”

Male, 50

"Some of the activities I had never tried. I’ve done a lot of counselling and psychology and stuff but I hadn’t actually tried those methods so they were quite useful."

Female, 56

"It works well in that it’s a prompt to get you to think about things.”

Male, 42

The main criticisms of the myCompass program related to connectivity issues, including slow downloading speed, and difficulties understanding program features due to insufficient instructions.

"I pretty much went on there every day, until I just got frustrated with it because it was taking so long."

Male, 63

"There were too many names of things, you know. It confused me.”

Female, 41

When asked about the usefulness of myCompass for people with diabetes, many users reported noticing improvement in their mood over the intervention period and acknowledged that myCompass may be a useful first step to accessing mental health support for people with diabetes.

"If you really didn’t want to go and do anything else, it would be quite suitable for the short-term. However you may then need to talk to someone professional.”

Male, 60

Some participants also reported an indirect effect of the program on their ability to manage their diabetes.

"It helped with my mood, and if everything was calm the sugars were much easier to control."

Male, 50

## Discussion

### Principal Findings

Depressive symptoms were significantly reduced in people with diabetes after using the myCompass intervention for 7 weeks. The effect size at postintervention was large (*d*=−1.28), with treatment gains maintained for 3 months. Significant and persistent improvements were also seen in anxiety symptoms, work and social functioning, and mental health self-efficacy. These preliminary data concur with previous controlled investigations of myCompass [16,17] and Internet-delivered treatments generally [44-47] and are in line with findings for guided disease-specific interventions and face-to-face CBT in people with diabetes [48,49]. Satisfaction with myCompass was in line with the community sample [16], with convenience and ease of program use and relevance of program content rated positively by participants. Psychotherapy delivered via mobile phone and Web technology without human support seemingly shows promise as an effective and acceptable treatment option for people with diabetes.

Interestingly, we also found significant and sustained improvements (with large effect sizes) in diabetes self-efficacy beliefs and diabetes-related distress; results that are striking considering that myCompass is a public health intervention with no diabetes-specific content. Of course, depressive symptoms correlate strongly with these cognitive variables [50], and treating depression alone may reduce the perceived demandingness of the disease and improve self-confidence (by reducing the negative bias that characterizes information processing in depression [51]). Alternatively, myCompass may have a direct effect on psychological constructs that underlie both depressive symptoms and diabetes-related distress (eg, emotional distress; [22]), and/or core cognitive and behavioral skills that are beneficial for people with one or both conditions. The mechanisms whereby a generic intervention produces diabetes-specific benefits need to be explored further. Nevertheless, our preliminary efficacy data suggest that a fully automated and generic public health intervention may have therapeutic benefits for patients with diabetes that extend beyond common mental health symptoms to include clinically important disease-specific cognitive, behavioral (eg, self-care) and physical symptom (eg, hyperglycemia) outcomes.

### Implications

International guidelines emphasize the importance of identifying and addressing emotional problems in the context of diabetes care [52], yet comorbid depression and distress are frequently untreated. Development of diabetes-specific Web-based interventions is an emerging paradigm that offers a possible solution [11-13], nevertheless, competition for funding for development and testing of eHealth solutions is fierce. At the same time, urgent action is required to immediately improve the well-being of people with diabetes and reduce the broader economic impact of depression in this high-risk group. Our findings suggest that myCompass, a public health intervention that is already broadly available and deliverable at minimal cost [53], may be a clinically and cost-effective and timely treatment option; a possibility that we are investigating more rigorously in a recently commenced placebo-controlled study.

At the primary care level, the opportunity to prescribe to patients a generic, low-intensity, and readily available psychotherapeutic intervention is likely to assist practitioners who encounter depressive symptoms within the context of multimorbidity [14,15], have difficulty discussing mental health issues with their diabetic patients (for fear of inadvertently making matters worse [52]), and/or struggle to disentangle depressive from diabetes-specific emotional and physical symptoms [15,54]. Furthermore, because myCompass can be efficiently disseminated to GPs in a single education session (as opposed to multiple sessions for different disease-specific programs), it is potentially an attractive, realistic, and cost-effective training option for practitioners negotiating the competitive demands and time pressures of primary care [55].

Privacy and stigma concerns can be major barriers to help seeking, and there are many people with diabetes who avoid confiding in health professionals for fear that their efforts and experiences will be misunderstood or patronized [56,57]. Others talk openly about their problems with their GP, but nevertheless refuse a referral for ongoing face-to-face care [37]. For these groups, a fully automated self-help program that is private, free to use, available 24/7, and able to screen symptoms and provide a tailored intervention may be a more palatable treatment alternative.

### Limitations

Caution is required in attributing symptom improvements to the intervention as our uncontrolled findings may reflect the natural course of symptom remission [58], or perhaps even regression toward the mean. Nevertheless, the improvements observed in this study are similar to those of controlled studies of Internet-delivered interventions (including myCompass; [16]), and reviews suggest a time course for recovery from mild-to-moderate depression of between 4 and 12 months [58,59]. Further study is required to clarify whether myCompass accelerates symptom alleviation for people with diabetes.

As data were derived from an older, highly educated, and predominantly female group of community volunteers, our findings may not generalize to other diabetic patient groups, including young people with T1D (a group at particularly high risk of mental health problems [60]), and patients recruited in primary care clinics. Consistent with other studies of self-guided Internet-delivered interventions, study attrition was also high (eg, [61-63]). Although our statistical methods accounted for dropout attrition and noncompletion, the potential for attrition bias remains. For example, it is not clear whether dropouts; (1) were less satisfied with the intervention; (2) experienced fewer symptom benefits than nondropouts, or (3) experienced symptom improvements early and dropped out because they no longer believed the intervention was required. Our placebo-controlled study will help shed light on relations between program usage, symptom outcomes, and attrition rates.

Finally, the large number of outcomes resulted in numerous statistical tests and possible inflation of the type 1 error rate (ie, mistakenly claiming significant effects in their absence). However, feasibility and exploratory studies generally have higher tolerance for type 1 errors [64], and most of our significant findings would have remained as such had more stringent control of the type 1 error rate been applied.

### Conclusions

Depressive symptoms were reduced in people with diabetes after use of a fully automated, mobile phone, and Web-based, public health intervention that is freely available. Importantly, significant and sustained improvement was also observed in comorbid anxiety and functional impairment with treatment gains extending to clinically relevant diabetes-specific cognitions, behaviors, and physical symptoms. Although an uncontrolled study, these preliminary results are encouraging and warrant further controlled investigation. myCompass shows promise as an intervention for depression and mental health comorbidities in people with diabetes and may overcome accessibility difficulties and other barriers to help seeking for people who might otherwise not access the psychological support they need.

## Abbreviations

CBT: cognitive behavior therapy
DSES: Diabetes Self-efficacy Scale
T1D: type 1 diabetes
T2D: type 2 diabetes
GP: general practitioner
LMM: linear mixed modeling
PAID: Problem Areas in Diabetes scale
PHQ-9: Patient Health Questionnaire
SD: standard deviation
SMS: short message service
WSAS: Work and Social Adjustment Scale
